# Prevalence of anemia and associated factors among pregnant women at Hargeisa Group Hospital, Somaliland

**DOI:** 10.1186/s12884-024-06539-3

**Published:** 2024-05-09

**Authors:** Mohamed Mussa Abdilahi, Jonah Kiruja, Badra Osman Farah, Farduus Mohamed Abdirahman, Ahmed Ismail Mohamed, Jama Mohamed, Abdeta Muktar Ahmed

**Affiliations:** Present Address: College of Medicine and Health Science, University of Hargeisa, Hargeisa, Somaliland

**Keywords:** Anemia, Pregnant women, Somaliland

## Abstract

**Background:**

Anemia remains a major global public health issue, affecting around 24.8% of the world’s population in both developing and developed countries. Pregnant women in developing countries are particularly susceptible, with 38.2% affected worldwide. Anemia is also a major contributor to maternal mortality, with 510,000 maternal deaths globally, of which 20% occur in developing countries and are related to anemia. Iron deficiency anemia is the most prevalent form, impacting 1.3 to 2.2 billion individuals, with 50% being women of reproductive age.

**Aim:**

This study aimed to assess the prevalence and associated factors of anemia in pregnant women attending antenatal care (ANC) at Hargeisa Group Hospital (HGH), Somaliland.

**Methods:**

A cross-sectional study included 360 pregnant women, who sought ANC at HGH from July 15 to August 6, 2023. The study subjects were selected using systematic random sampling. Data were collected through structured questionnaires and participants’ current medical charts, including hemoglobin levels. Data analysis was performed using SPSS software (version 20).

**Results:**

The study revealed an overall prevalence of anemia among pregnant women at 50.6% (95% CI: 45.40 − 55.72%). Anemia severity was categorized as mild (33.0%), moderate (54.9%), and severe (12.1%). Factors statistically associated with anemia included gestational age in the third trimester (AOR = 3.248, 95% CI: 1.491–7.074), lack of ANC visits (AOR = 6.828, 95% CI: 1.966–23.721), and absence of iron supplementation (AOR = 29.588, 95% CI: 2.922–299.713). Notably, a higher consumption of meat per week was associated with a reduced risk of anemia (AOR = 0.198, 95% CI: 0.104–0.379).

**Conclusion:**

The study underscores the severity of anemia in pregnant women within the range considered as severe public health problem by WHO. It is crucial to emphasize effective prenatal care, improve dietary practices, and promote the provision of iron supplements. Enhanced maternal education on Anemia during ANC visits has the potential to reduce Anemia prevalence and mitigate adverse maternal and neonatal outcomes.

## Background of the study

Anemia during pregnancy is defined by the Centers of Disease Control and Prevention (CDC) and the World Health Organization (WHO) as having a lower hemoglobin (Hgb) level than the normal range, leading to a reduced oxygen-carrying capacity of red blood cells to body tissues. WHO classifies Anemia into different severity levels based on hemoglobin levels: mild (10–10.9 g/dl), moderate (7–9.9 g/dl), and severe (< 7.0 g/dl**)** [1]. In terms of prevalence, Anemia’s severity, according to WHO, is categorized as mild (prevalence between 5% and 20%), moderate (prevalence between 20% and 40%), and severe (prevalence greater than 40%) [2].

Anemia is a major public health concern, particularly in low and middle-income countries, affecting 1.62 billion people, or 24.8% of the world’s population [3]. It is worth noting that 32.4 million pregnant women (38.2%) worldwide experience Anemia during pregnancy [1].

Annually, approximately 510,000 maternal deaths occur due to complications related to pregnancy and childbirth, with nearly 20% of these deaths occurring in developing countries, often attributable to Anemia [1]. The prevalence of Anemia in developing and developed countries is estimated at 43%, whereas high-income countries have a prevalence of 9%. Anemia contributes to over 115,000 maternal deaths per year [4].

In low and middle income countries, Anemia in pregnant women is primarily related to iron deficiency, accounting for 75% of Anemia cases. This deficiency results from inadequate dietary iron intake or absorption, increased iron requirements during pregnancy, iron losses due to blood loss, worm infestations, infections, and low health-seeking behaviors [5]. Anemia is a leading cause of maternal and Fetal complications, resulting in prolonged labour, premature births, low birth weight, cognitive impairment, and even death [4, 6].

To combat Anemia, it is recommended to provide intermittent iron and folic acid supplementation for women of reproductive age group living in areas with a prevalence of Anemia of 20% or higher. Pregnant women should receive daily iron and folic acid supplementation as part of antenatal care (ANC) to prevent Anemia during pregnancy [7].

According to a WHO report, the prevalence of Anemia among pregnant women is 41.8%, with the highest prevalence (61.3%) in Africa and 52.5% in South East Asia. Sub-Saharan Africa (SSA) is the most affected region, with 17.2 million pregnant women, accounting for 30% of the total global cases [6, 8].

In Somaliland, as reported by the Somaliland Demographic Health Survey, only 47% of women of reproductive age receive ANC services from skilled providers, such as doctors, nurses or midwives. Somaliland grapples with one of the world’s highest maternal mortality rates (396 per 100,000 live births) [9].

Globally, about 50% of Anemia is caused by iron deficiency [4]. Notably, despite the fact that 62% of women of childbearing age in Somaliland do not take the recommended number of folic acid supplements during their pregnancy, there has been no prior study to determine the prevalence of Anemia among pregnant women in Somaliland [9].

Therefore, this study aims to determine the prevalence of Anemia and assess associated factors among pregnant women attending ANC at Hargeisa Group Hospital (HGH) in Somaliland. The findings from this study can serve as guidance for ANC service providers and other stakeholders working to address this issue. Additionally, it can serve as a baseline dataset for researchers interested in this area.

## Methods and materials

### Study design and setting

The institutional-based cross-sectional study design was conducted to assess the prevalence and associated factors of anemia among pregnant women attending at HGH from July 15 to August 6, 2023. HGH is located in Hargeisa, the capital city of Somaliland, and comprises nine sub-administrative districts. The hospital serves a population of over 1.5 million people, making it a significant healthcare facility in the region. On average, the ANC unit at HGH receives approximately 30 pregnant women per day.

### Study population, inclusion, and Exclusion Criteria

The study population included all pregnant women who received ANC at HGH during data collection period. Pregnant women who were physically present at the outpatient department/ANC clinic during data collection were included in this study, while pregnant women who had previously visited the ANC unit during the study period were excluded.

In Somaliland, pregnant women have access to free ANC services in all government health facilities starting from the first month of pregnancy. These services encompass a comprehensive range of care, including folic acid and iron supplements, as well as laboratory tests to monitor the hemoglobin levels of pregnant women [9].

### Measurement of variables

#### Outcome variable

The outcome variable in this study is Anemia. According to the WHO categories for hemoglobin concentration values, pregnant mothers were grouped into one of four groups: no Anemia (hemoglobin ≥ 11.0 g/dl), mild Anemia (hemoglobin 10.0–10.9 g/dl), moderate Anemia (hemoglobin 7.0–9.9 g/dl) and severe Anemia (hemoglobin < 7.0 g/dl).

#### Independent variables

##### Socio-demographic variables

The socio-demographic variables include women’s age (categorized as ≤ 20 years, 21–29 years, and ≥ 30 years), educational status (categorized as literate or illiterate), occupation (categorized as employed or unemployed) and family size (categorized as 1–3 households and > 4 households).

##### Obstetric variables

The obstetric variables Include Gravidity (categorized as multigravida and Primigravidae), pregnancy trimester (categorized as first, second, and third), birth spacing (categorized as < 2 years and ≥ 2 years), ANC visit during the current pregnancy (categorized as yes or never visited), type of current pregnancy (categorized as single or twins) and iron supplementation during the current pregnancy (categorized as yes or no).

##### Dietary variables

The dietary variables include consumption of tea or coffee immediately after a meal (categorized as yes or no), frequency of daily meat consumption (categorized as more than once per week or less than once per week) fruit and vegetable consumption (categorized as more than once per day or less than once per day), and milk consumption (categorized as more than once per day or less than once per day).

### Sample size determination

To ascertain the sample size for this study, we employed the single population proportional formula. This calculation was based on several parameters, including a 95% confidence level, a    5% margin of error, and an estimated proportion (P) derived from a previous study (26.7%) [10]. To account for potential non-responses, a non-response rate of 10% and additional 10% for possible incomplete medical chart was included in the calculation. The single population proportional formula used for sample size determination is as follows:

$$n=\frac{{{Z^2}P(1 - P)}}{{{E^2}}}$$

Where:

*n* is the required sample size.

*Z* is the Z-score corresponding to the chosen confidence level (for a 95% confidence level, *Z* ≈ 1.96).

*P* is the estimated proportion of Anemia (26.7%).

*E* is the margin of error (10%).

Using this formula, the initial estimated sample size was calculated as 300 participants. However, to ensure the study’s robustness and representativeness, we adjusted the sample size to 360 participants, considering the non-response and possible incomplete medical rate. This larger sample size provided a more comprehensive and accurate reflection of the pregnant women attending HGH.

### Sampling technique and procedure

The study employed a systematic random sampling technique. To establish the sample, we first determined the average number of pregnant women attending the ANC clinic (outpatient department) over the previous month, which was found to be 720. To ensure a representative sample, we calculated a sampling fraction of 2 (*N*/*n*) by dividing the total number of pregnant women who received ANC in the last month by the required sample size.

Subsequently, we initiated the sampling process by selecting the first participant randomly through a lottery method. Thereafter, we included every second pregnant woman from this initial group. However, in instances where the chosen participant did not meet the inclusion criteria or declined to participate, the subsequent participant in line was considered, ensuring that the sample maintained its intended size and representativeness. This approach allowed us to efficiently gather data from the pregnant women in the ANC clinic at HGH while ensuring randomization and coverage of the required sample size.

### Data collection tools and procedure

Data collection was carried out using structured questionnaires designed specifically for this study. The questionnaire was adapted from relevant published literature and then tailored to the local context to ensure the collection of pertinent information about pregnant women [6, 11]. Questions comprised socio-demographic characteristics, such as age, educational background, occupation, and family size, as well as obstetric and dietary related factors, including gravidity, pregnancy trimester, birth spacing, ANC attendance, type of pregnancy, and dietary practices. In addition to questionnaire-based data, information regarding the hemoglobin levels of the participants was retrieved by reviewing their medical charts during the data collection process. This multi-faceted data collection approach allowed for a comprehensive understanding of the factors related to anemia among pregnant women. The data collection process was conducted by a team of two senior midwives and one experienced supervisor, ensuring the quality and accuracy of the data obtained.

### Data quality assurance

Before commencing the actual data collection process, a preliminary pretest was conducted on 10% of the anticipated sample size and additional 10% for possible incomplete medical chart. This pre-test took place at a location outside of the study area, specifically at the Mohamed Mooge Health Centre. The primary objectives of this pre-test were to evaluate the comprehensiveness, effectiveness, reliability, and validity of the data collection tool. Following the pre-test, minor adjustments were made to the wording and format of the questionnaire to enhance its suitability for efficient data collection.

To maintain data quality and accuracy, the data collectors and their supervisor underwent a comprehensive one-day training session. This training was conducted to ensure that all team members were well-versed in the study’s objectives and the appropriate data collection procedures.

During the data collection phase, the filled questionnaires were meticulously reviewed on a daily basis by both the supervisor and the principal investigator. This stringent review process aimed to verify the completeness and accuracy of the collected data. If any discrepancies or issues were identified, the necessary feedback and guidance were provided to the data collectors for immediate resolution.

Furthermore, to uphold participant confidentiality and privacy, all eligible participants who voluntarily consented to participate in the study were interviewed in private settings. This approach was adopted to create a secure and comfortable environment for the participants, thereby promoting candid and honest responses while safeguarding their personal information.

### Data analysis

The statistical analysis for this study was conducted using the Statistical Package for the Social Sciences (SPSS), specifically version 20. To present the demographic information and other variables, descriptive statistics such as frequency and percentage were employed. Additionally, data were illustrated using tables, graphs, and various other mechanisms for data summarization.

Both bivariate and multivariate analyses were carried out to explore the relationships and associations within the data. In the bivariate analysis, variables with a *P*-value equal to or less than 0.05 were selected for further investigation in the multivariate logistic model. This multivariate analysis aimed to identify variables of significance while controlling for potential confounding factors.

In measuring associations, Adjusted Odds Ratios (AORs) were utilized, accompanied by a 95% Confidence Interval (CI). Variables with a *P*-value equal to or less than 0.05 were considered statistically significant, signifying a robust relationship or effect within the context of the study.

## Results

### Socio-demographic factors of the study participants

In this study, a total of 360 participants were included. Examining the age distribution, it became evident that 85 individuals (46.7%) fell within the age range of 21 to 29 years, and this group exhibited a higher prevalence of Anemia during their pregnancy compared to those who were 20 years old or younger (25 individuals, 13.7%).

Furthermore, the analysis revealed that the majority of anaemic participants (93 individuals, 51.1%) belonged to families with a larger size, consisting of more than four members, as opposed to those residing in smaller households (89 individuals, 48.9%). The educational status of the participants was also an important factor, as the vast majority (156 individuals, 85.7%) who had developed Anemia were found to be illiterate. In addition, an overwhelming majority (172 individuals, 94.5%) of those with Anemia were unemployed (Table 1).

**Table 1:** Socio-demographic factors of pregnant women (*n* = 360)

Variables	Anemia		Total (%)	*P*-value
	Yes *n* = 182%	No *n* = 178%	*n* = 360%	
Age of the mother				
≤ 20 years old 21–29 years old ≥ 30 years old	25(13.7%) 85(46.7%) 72 (39.6%)	23 (12.9%) 110(61.8%) 45(25.3%)	48 (13.3%) 195 (54.2%) 117 (32.5%)	0.009*
Family size				
1–3 ≥ 4	89 (48.9%) 93 (51.1%)	87 (48.9%) 91 (51.1%)	176 (48.9%) 184(51.1%)	0.996
Educational level				
Literate Illiterate	26 (14.3) 156 (85.7%)	30 (16.9%) 148 (83.1%)	56 (15.6%) 304 (84.4%)	0.501
Occupational level				
Employed Unemployed	10(5.5%) 172(94.5%)	14(7.9%) 164(92.1%)	24(6.7%) 336(93.3%)	0.367

Note: *Statistically significant, *P*-value of ≤ 0.05

### Obstetric factors of the study participants

The findings pertaining to obstetric factors among pregnant women shed light on significant trends. It is notable that the vast majority, consisting of 145 (79.74%) of the pregnant women, were multigravida and had experienced Anemia during their pregnancies. Another compelling revelation was related to birth intervals. Approximately two-thirds, specifically 108 (59.3%) of the participants with birth intervals of less than two years, were reported to be anaemic. When analyzing the current pregnancy status, it was apparent that a greater majority of the respondents, numbering 176 (96.7%), who were carrying a single fetus, had developed Anemia. The trimester of pregnancy also played a role in Anemia occurrence. A total of 102 (56%) participants who were in their third trimester were identified to have Anemia. Furthermore, it was noted that a majority of eligible participants, totalling 177 (97.3%), had never received ANC follow-up. Lastly, the majority, comprising 181 (99.5%), of the participants who had developed Anemia had never taken iron supplements (Table 2).

**Table 2:** Obstetric factors of pregnant women (*n* = 360)

Variables	Anemia		Total (%)	*P*-value
	Yes	No		
Gravidity				
Multigravida Primigravidae	145 (79.7%) 37(20.3%)	128 (71.9%) 50 (28.1%)	273(75.8%) 87(24.2%)	0.086
Birth interval				
≤ 2 years ≥ 2 years Not applicable	108 (59.3%) 36 (19.8%) 38(20.9%)	100 (56. 1%) 27 (15.2%) 51(28.7%)	208(57.8%) 63(17.5%) 89(24.7%)	0.178
Current pregnancy				
Single Twins	176 (96.7%) 6(3.3%)	169 (94.9%) 9(5.1%)	345(95.8%) 15(4.2%)	0.404
Gestational Age				
First trimester Second trimester Third trimester	17 (9.4%) 63(34.6%) 102(56%)	32(18%) 64 (36%) 82(46)	49(13.6%) 127(35.3% 184(51.1%)	0.035*
ANC fallow up				
Yes No	5(2.7%) 177 (97.3%)	96 (53.9%) 82(46.1%)	101(28.1%) 259 (71.9%)	0.000*
Iron supplements				
Yes No	1 (0.5%) 181 (99.5%)	87(48.9%) 91 (51.1%)	88(24.4%) 272(75.6%)	0.000*

Note: *Statistically significant, *P*-value of ≤ 0.05

### Nutrition-related 0characteristics of the study participants

Table 3 shows the findings concerning the nutrition-related characteristics of pregnant women. Among the participants who had Anemia, a significant majority of 162 (89%) reported that they consumed meat more than once per week. Moreover, approximately 123 (67.6%) of pregnant women who consumed fruits and vegetables more than once per day were found to be anaemic. The current study also highlighted that 171 (64.3%) of pregnant women who consumed milk more than once per day were anaemic. Furthermore, the analysis revealed that a significant portion, specifically 108 (59.3%), of pregnant women who had never consumed tea or coffee immediately after a meal were anaemic (Table 3).

**Table 3:** Nutrition-related characteristics of pregnant women (*n* = 360)

Variables	Anemia		Total (%)	*P* value
	Yes	No		
How often do you eat meat?				
More than once per week Less than once per week	162(89%) 20(11%)	106 (59.6%) 72(40.4%)	268(74.4%) 92(25.6%)	0.000*
How often do you eat fruits and vegetables?				
More than once per day Less than once per day	123(67.6%) 59(34.4%)	96(53.9%) 82(46.1%)	219(60.8%) 141(39.2%)	0.008*
How often do you drink milk?				
More than once per day Less than once per day	171(64.3%) 65(35.7%)	92(51.7%) 86(48.3%)	209(58.1%) 151(41.9%)	0.015*
Do you drink tea/coffee immediately after meal?				
Yes No	108(59.3%) 74 (40.7%)	58 (32.6%) 120(67.4%)	166(46.1%) 194(53.9%)	0.000*

Note: *Statistically significant, *P*-value of ≤ 0.05

### Prevalence and severity of Anemia among pregnant women

**Fig. 1:**
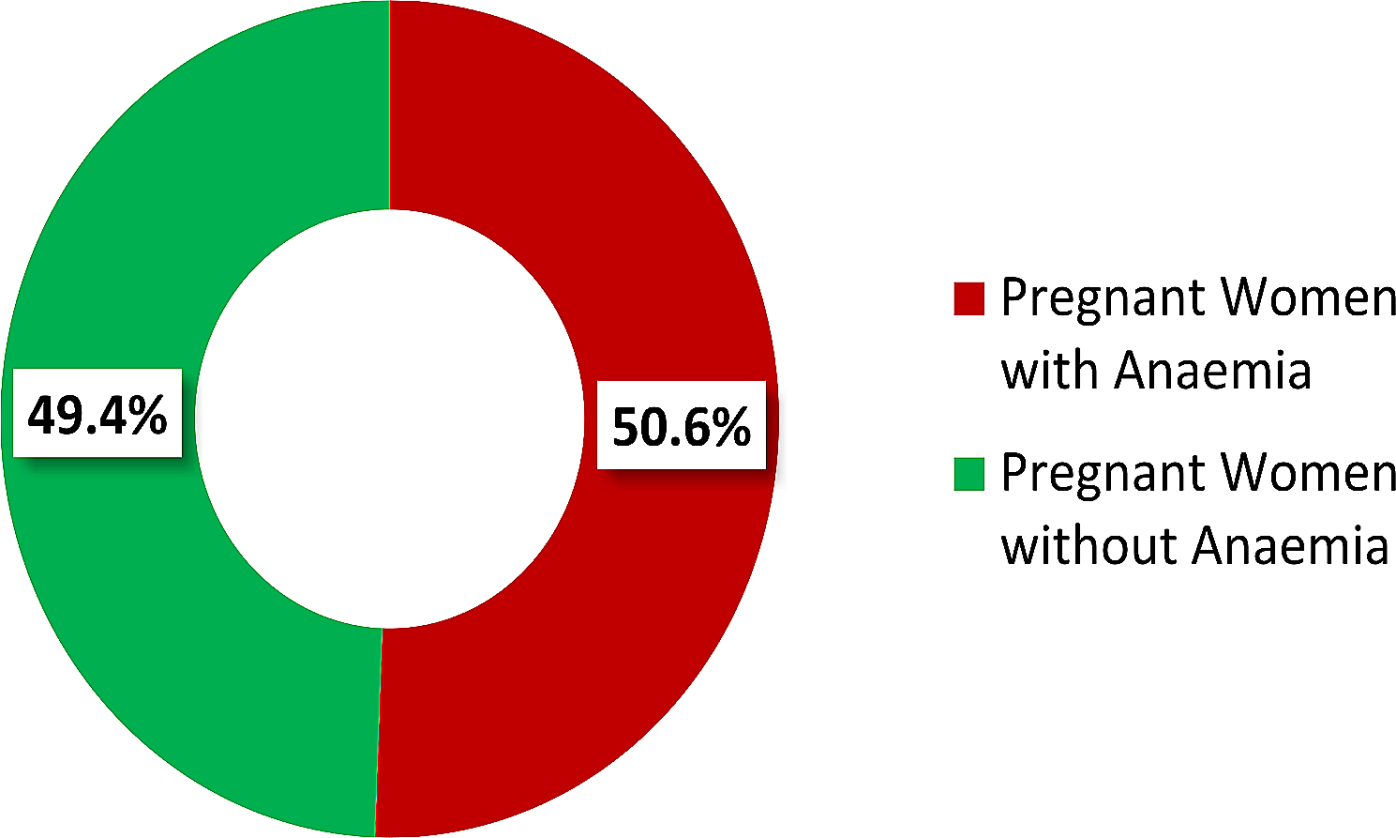
Prevalence of anaemia among pregnant women attending ANC at HGH, Hargeisa Somaliland, 2023

The study revealed that the overall prevalence of Anemia among pregnant women attending HGH, using a cut off level of hemoglobin < 11 g/dl (< 33% Haematocrit), was found to be 50.6% [182 out of 360, 95% CI: (45.40 − 55.72%)], as illustrated in Fig. 1.

**Fig. 2:**
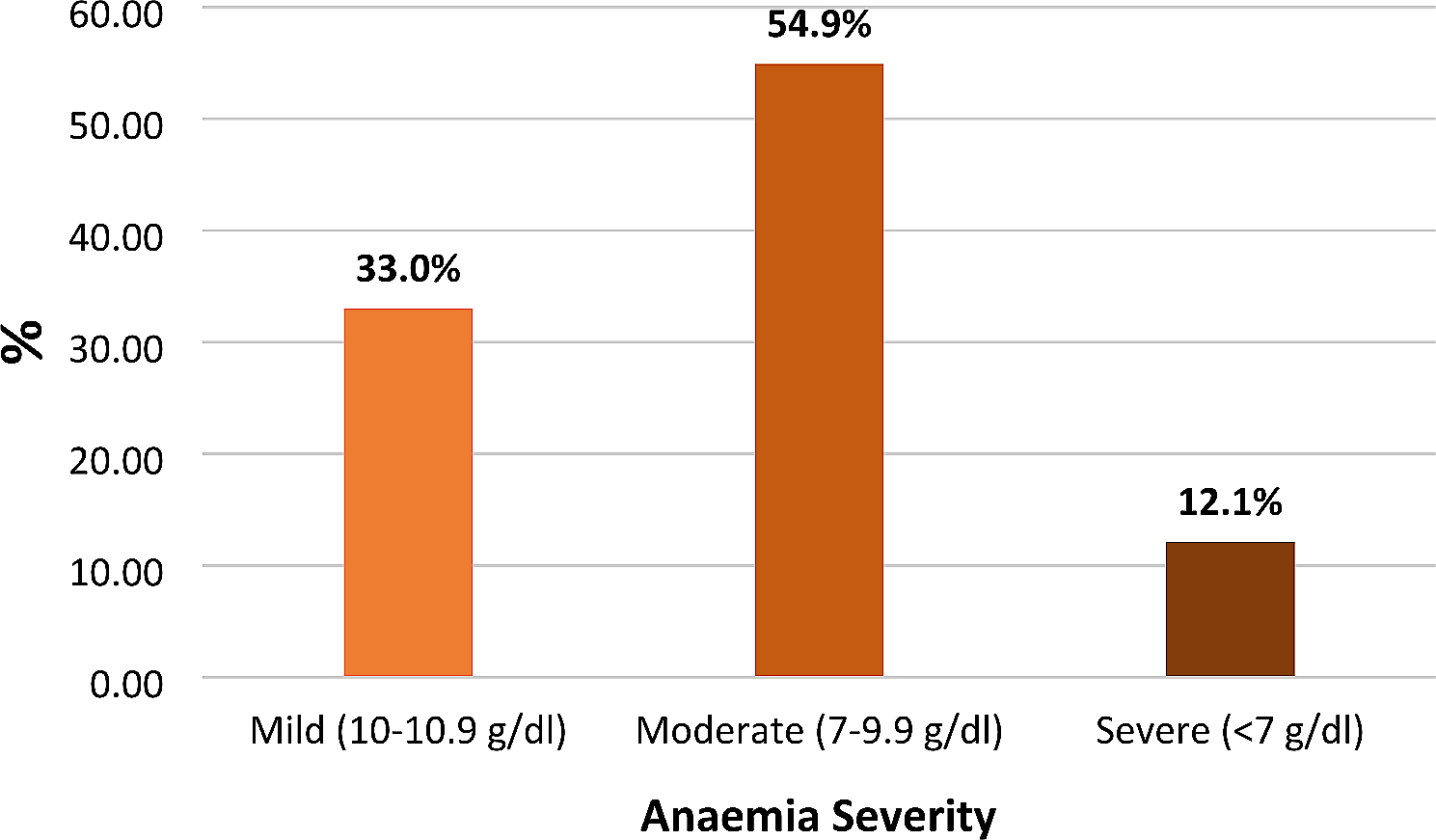
Distribution of the severity of anaemia among pregnant women receiving ANC at HGH, Hargeisa, Somaliland, 2023

Among the anaemic pregnant women, approximately 12.1% (22 out of 182) were classified as severely anaemic, while 54.9% (100 out of 182) fell into the category of moderate Anemia, and 33.0% (60 out of 182) exhibited mild Anemia, as exhibited in Fig. 2.

### Factors associated with prevalence of Anemia among pregnant women

In the bivariate model, six variables exhibited a P-value below 0.05, making them eligible for inclusion in the multivariable regression model. However, upon multivariate analysis, only four independent variables demonstrated statistical significance. These significant factors were gestational age, ANC follow-up of pregnant women, iron supplement intake, and meat consumption per week.

The likelihood of developing Anemia among participants in the third trimester of gestational age was approximately three times higher [AOR = 3.248 (95% CI: 1.491, 7.074)] compared to those in the first trimester. Pregnant women who had never attended ANC check-ups had 6.828 times higher odds of experiencing Anemia [AOR = 6.828 (95% CI: 1.966, 23.721)] compared to women with regular ANC follow-up. Similarly, the study revealed that the risk of Anemia was significantly higher among pregnant women who had never taken iron supplements, with odds approximately 29 times higher [AOR = 29.588 (95% CI: 2.922, 299.713)] compared to those who used iron supplements. Additionally, the analysis showed that the odds of developing Anemia decreased by 80% among pregnant women who consumed meat more frequently (At least once per week) [AOR = 0.198(95% CI: 0.104, 0.379)] compared to their counterparts. The details are shown in Table 4.

**Table 4:** Factors Associated with prevalence of anaemia among pregnant women

Variables	Anemia		COR (95%CI)	AOR (95%CI)
	Yes	No		
**Gestational Age**				
First trimester Second trimester Third trimester	17 (9.4%) 63(34.6%) 102(56%)	32(18%) 64 (36%) 82(46)	1 2.075(0.872–4.940) 2.400(1.052–5.476)	1 1.250(0.681–2.292) 3.248(1.491–7.074) *
**ANC fallow up**				
Yes No	5(2.7%) 177 (97.3%)	96 (53.9%) 82(46.1%)	1 5.822 (1.527–22.199)	1 6.828(1.966–23.721) *
**Iron supplements**				
Yes No	1 (0.5%) 181 (99.5%)	87(48.9%) 91 (51.1%)	1 40.561 (3.799-433.013)	1 29.588 (2.922-299.713) *
**Eat meat per week**				
At least once per week Less than once per week	162(89%) 20(11%)	106 (59.6%) 72(40.4%)	0.199 (0.101–0.393) 1	0.198 (0.104–0.379) * 1

Note: *Statistically significant, *P*-value of ≤ 0.05

## Discussion

This study aimed to assess the prevalence of Anemia and its associated factors among pregnant women receiving ANC at HGH. In this study, 50.6% of pregnant women were found to have Anemia. According to WHO classification, this prevalence of Anemia among pregnant women suggests a severe public health concern [12]. This finding aligns with other studies conducted in countries such as India (50.9%) and Egypt (51.3%) [13, 14], but lower than findings from studies conducted in Ghana (56%), Bangladesh (62.5%) and Pakistan (57.7%) [15–17]. Conversely, it is much higher than findings from studies in Yemen (25%), Somalia (44.4%), Ethiopia (7.9%), Tanzania (25.5%) and Saudi Arabia (34.1%) [3, 5, 6, 12, 18]. The variation might be attributed to geographical differences, characteristics of the target population, and differing interventions to address Anemia in pregnancy across settings.

In this study, Anemia during pregnancy was higher among multigravida women (79.74%) compared to primigravidae. This is much higher than studies conducted in Pakistan (54.2%) and Uganda (29.1%) [17, 19]. The increased prevalence of Anemia with increased gravidity might be due to the repeated births and high fertility behaviour, which is a risk factor for micronutrient depletion owing to high demand and frequent blood loss during childbirth, ultimately affecting micronutrient storage. This observation highlights the potential risk for Anemia among women with multiple pregnancies.

Additionally, the prevalence of Anemia was highest (56%) during the third trimester of pregnancy, higher than a study among pregnant women in Uganda (24.4%) [19]. The higher incidence of Anemia during the third trimester may be attributed to insufficient ANC and low iron supplement intake, possibly due to the greater iron demand compared to the first and second trimesters.

Pregnant women in the third trimester (AOR = 3.248, 95% CI: 1.491, 7.074) were significantly associated with the occurrence of anemia, consistent with findings from Ethiopia (AOR = 4.9, 95% CI: 1.39, 17.6) [6]. This increased incidence of Anemia during the third trimester may also indicate inadequate ANC, iron supplementation, and nutrition.

Our findings further reveal that pregnant women who did not visit ANC during pregnancy (AOR = 6.828, 95% CI: 1.966, 23.721) were significantly associated with the occurrence of anemia, in line with a study conducted in Somalia (AOR = 6.707, 95% CI: 1.390, 32.352) [5]. The provision of micronutrients (such as folic acid and iron), nutrition education, and related care during ANC visits may play a role in reducing the risk of Anemia. The lack of ANC follow-up can be seen as a contributing factor to the prevalence of Anemia among this population.

Women who had no iron supplements regularly (AOR = 29.588, 95% CI: 2.922, 299.713) were significantly associated with the occurrence of anemia. This study is consistent with findings from systematic review and Meta-analysis that was done in sub-Saharan Africa (OR: 1.82, 95% CI: 1.22, 2.70) [20]. This association may be explained by the increased iron requirements during pregnancy. This finding highlights the potential role of iron supplementation in preventing Anemia among pregnant women.

A higher consumption of meat per week (AOR = 0.198, 95% CI: 0.104, 0.379) were significantly associated with a reduced risk of Anemia, in contrast to another study conducted in Somalia (AOR = 19.595, 95% CI: 2.264, 169.606) [5]. This suggests that a decrease in meat intake during pregnancy is associated with increased risk of Anemia, likely due to its role as a crucial dietary source of heme iron. This observation raises questions about the adequacy of meat consumption in relation to Anemia prevention.

## Limitation of the study

While this study aimed to assess the prevalence of Anemia and its associated factors among pregnant women, it is important to acknowledge several limitations. First, it should be noted that this study was institution-based, and the anaemic women included in the study were a sample of those who visited the hospital. As a result, the findings may not be fully representative of the entire population of pregnant women in Somaliland. To address this limitation, future research should consider conducting community-level studies to strengthen the generalizability of the results.

Secondly, this study lacks a qualitative component that could delve into the perceptions of women regarding the causes of Anemia and strategies for its prevention during pregnancy. Including qualitative insights would provide a more comprehensive understanding of the issue.

Additionally, the study did not account for certain factors, such as parasitic infections and genetic disorders, which can play a significant role in the development of Anemia. Future research should consider these factors for a more thorough analysis of anemia’s determinants.

Finally, it is important to acknowledge that the cross-sectional study design used in this research has limitations when establishing causal associations. Cross-sectional studies are better suited for identifying associations rather than causation due to their snapshot-in-time nature. Therefore, the findings should be interpreted with this limitation in mind.

## Conclusions

The prevalence of Anemia among pregnant women in the study area was found to be 50.6% (95% CI: 45.40 − 55.72%). This prevalence falls within the range considered a severe public health problem by WHO. Several independent risk factors for Anemia were identified, including the gestational age of the mother, ANC attendance, iron supplementation, and the frequency of meat consumption per week. To address this issue effectively, targeted efforts should focus on encouraging early antenatal attendance among women in these at-risk groups. This approach would enable timely iron and folic acid supplementation, provide dietary guidance, and deliver regular education on Anemia during pregnancy, potentially reducing the overall prevalence of Anemia.

## Data Availability

All the data sets used and/or analyzed during the current study are available from the corresponding author upon reasonable request.

## Abbreviations

ANC: Antenatal Care
AOR: Adjusted Odds Ratio
CI: Confidence Interval
COR: Crude Odds Ratio
HGH: Hargeisa Group Hospital
